# Determinants in HIV-2 Env and tetherin required for functional interaction

**DOI:** 10.1186/s12977-015-0194-0

**Published:** 2015-08-07

**Authors:** Colin M Exline, Su Jung Yang, Kevin G Haworth, Srinivas Rengarajan, Lisa A Lopez, Magali E Droniou, Eduardo Seclen, Paula M Cannon

**Affiliations:** Department of Molecular Microbiology and Immunology, Keck School of Medicine, University of Southern California, 2011 Zonal Avenue, HMR 502, Los Angeles, CA 90033 USA

## Abstract

**Background:**

The interferon-inducible factor BST-2/tetherin blocks the release of nascent virions from the surface of infected cells for certain enveloped virus families. The primate lentiviruses have evolved several counteracting mechanisms which, in the case of HIV-2, is a function of its Env protein. We sought to further understand the features of the Env protein and tetherin that are important for this interaction, and to evaluate the selective pressure on HIV-2 to maintain such an activity.

**Results:**

By examining Env mutants with changes in the ectodomain of the protein (virus ROD14) or the cytoplasmic tail (substitution Y707A) that render the proteins unable to counteract tetherin, we determined that an interaction between Env and tetherin is important for this activity. Furthermore, this Env-tetherin interaction required an alanine face in the tetherin ectodomain, although insertion of this domain into an artificial tetherin-like protein was not sufficient to confer sensitivity to the HIV-2 Env. The replication of virus carrying the ROD14 substitutions was significantly slower than the matched wild-type virus, but it acquired second-site mutations during passaging in the cytoplasmic tail of Env which restored the ability of the protein to both bind to and counteract tetherin.

**Conclusions:**

These results shed light on the interaction between HIV-2 and tetherin, suggesting a physical interaction that maps to the ectodomains of both proteins and indicating a strong selection pressure to maintain an anti-tetherin activity in the HIV-2 Env.

## Background

Tetherin/BST-2 is a multi-functional cellular protein that plays roles in cell membrane organization, as well as contributing to both the sensing and inhibition of enveloped virus replication [reviewed in 1]. Depending on the cell type, tetherin can be constitutively expressed or stimulated by interferon [2–5]. Tetherin localizes to lipid raft membrane microdomains, where it links to the actin cytoskeleton and helps to stabilize the apical actin network and microvilli in polarized cells [6, 7]. Tetherin also has antiviral properties, that were first described against HIV-1 [8, 9]. In HIV-1 infected cells, tetherin retains newly assembled virions at the cell surface which both reduces the production of cell-free virus [8, 10] and also promotes natural killer cell mediated antibody-dependent killing of infected cells [11–13]. Additionally, the human form of tetherin, and to a lesser extent chimpanzee tetherin, can act as pattern recognition receptors, since cross-linking of the protein by tethered virions or antibodies activates the NF-κB pathway and promotes entry into an antiviral state [14, 15].

Structurally, tetherin is a type 2 transmembrane glycoprotein, with a short cytoplasmic tail and membrane-spanning domain at its N-terminus, and a GPI anchor at its C-terminus [6]. These membrane anchors flank an extracellular coiled-coil domain that mediates tetherin–tetherin interactions and promotes the formation of parallel homodimers, which can be further organized into tetramers [16, 17]. Tetherin retains budding virions at the cell surface in an axial conformation, with the GPI anchors preferentially incorporated into virions and the transmembrane domains anchored in cellular membranes [18]. All three of the major structural features of the protein are required for its ability to inhibit virus release [8, 19, 20], although the actual sequences are not essential, and its function can be recapitulated in a wholly artificial tetherin construct [20].

Since tetherin presents a barrier to virus replication at multiple levels, it is not surprising that the primate lentiviruses have evolved several strategies to counteract its actions. Most SIVs use the Nef protein to block tetherin [21–25], in a mechanism based on intracellular sequestration via a direct physical interaction between Nef and tetherin’s cytoplasmic tail [26]. Alternatively, some SIVs such as SIVgsn use Vpu to counteract tetherin, and Vpu persists as the viral anti-tetherin factor in present day group M HIV-1 [8, 9, 23]. Here the mechanism is also predominantly through intracellular sequestration, combined with ubiquitination and endolysosomal degradation [27–32]. A direct physical interaction between Vpu and tetherin has also been reported, that maps to the trans-membrane domains of each protein [33, 34].

In HIV-2, which does not encode Vpu, the anti-tetherin factor is the Env protein [35–37]. HIV-2 Env has been reported to both interact with tetherin [37] and to remove it from the cell surface, leading to its concentration in a perinuclear compartment [29, 37, 38]. This interaction appears to be mediated by the extracellular domains of the two proteins since a chimeric Env comprising the extracellular domain of HIV-2 Env linked to the transmembrane and cytoplasmic domains of the non-functional HIV-1 Env is still able to antagonize tetherin [35]. Conversely HIV-2 Env can counteract a tetherin derivative substituted with the transmembrane and cytoplasmic domains of the transferrin receptor, but retaining the extracellular domain and GPI anchor of native tetherin [38]. In addition to a requirement for the extracellular domain of HIV-2 Env, a tyrosine based sorting motif in the cytoplasmic tail has also been shown to be required for anti-tetherin activity [37, 39].

In the present study, we sought to more fully map the determinants in tetherin and the HIV-2 Env that allow their interaction, and to investigate the impact of the loss of anti-tetherin activity on HIV-2 replication. Specifically, we asked whether there was a selective pressure for a virus that had lost the ability to antagonize tetherin following mutation of Env to re-acquire this function, and whether this would once again map to the Env protein.

## Results

### Interaction of HIV-2 Env and tetherin is required for tetherin antagonism

**Fig. 1:**
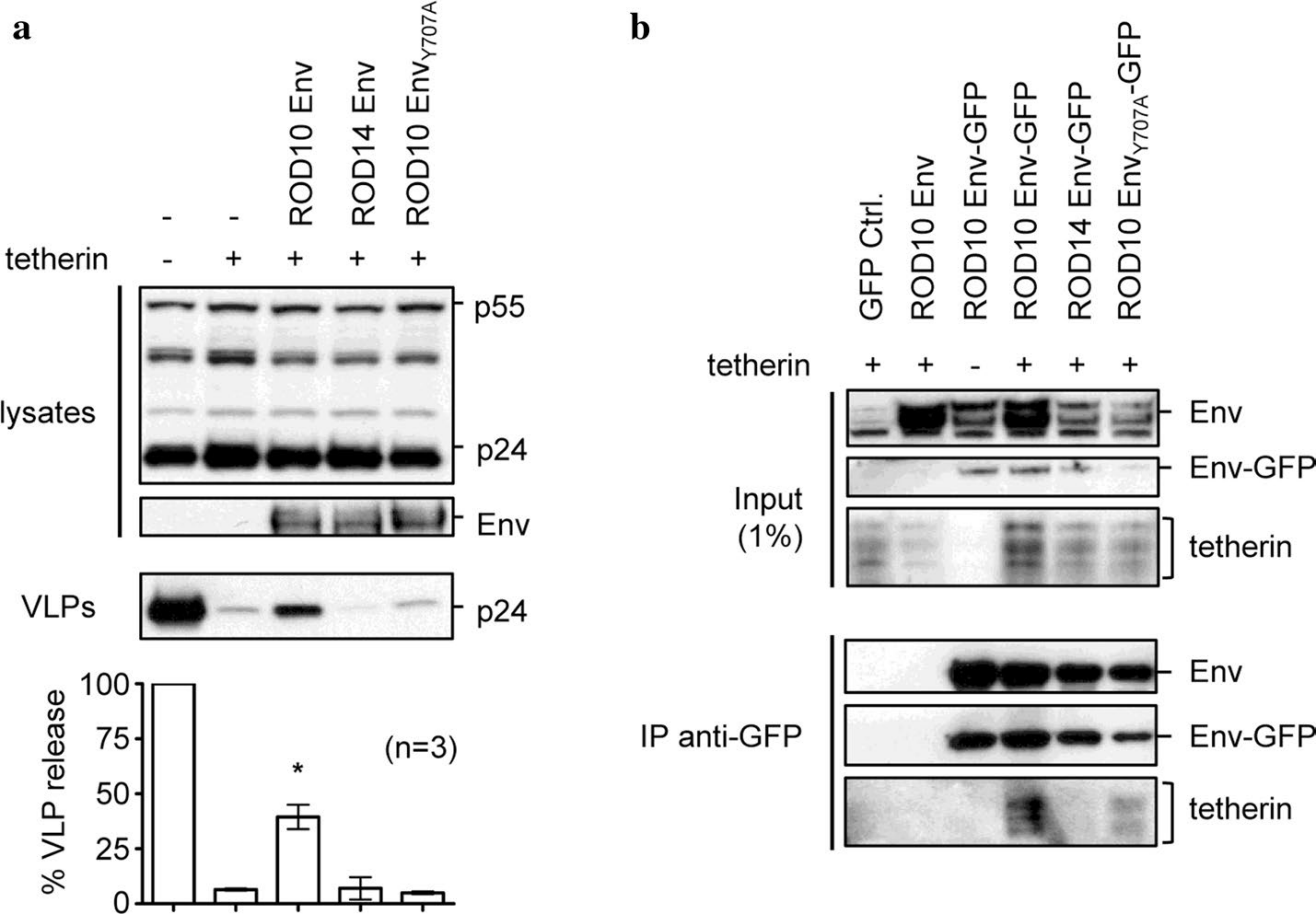
HIV-2 Env mutants that disrupt the interaction with tetherin. **a** HIV-1 Gag-Pol VLPs were created by transfecting 293A cells with pHIV-1-pack, together with a control CMV expression plasmid (−) or expression plasmids for tetherin and the indicated Envs. 24 h later, VLP release was measured as the ratio of p24-reacting bands in supernatants versus cell lysates following Western blot analysis. Results were normalized to the no tetherin control which was set at 100% VLP release. *Graphs* show mean plus standard deviation for n = 3 independent experiments, *p* < 0.05 (*). **b** 293T cells were co-transfected with tetherin and GFP-tagged Env variants. Cells were lysed 24 h later and GFP-tagged proteins were immunoprecipitated (IP) with anti-GFP MicroBeads, followed by Western blotting of the input lysates (1%) and IP products using anti-Env, anti-GFP or anti-tetherin antibodies.

Previously, it was reported that the Env protein from HIV-2 strain ROD10 can be co-immunoprecipitated with tetherin [37]. However it was not established if this interaction was necessary for the Env protein’s anti-tetherin activity. To analyze this further, we selected two closely related mutants of ROD10 Env that do not counteract tetherin [29, 37], and evaluated their ability to bind to the protein. The ROD14 Env differs from ROD10 Env at 5 specific amino acids, and contains a 30 amino acid deletion in its cytoplasmic tail [40], with substitutions K422R and A598T in the ectodomain of the protein being primarily responsible for its loss of tetherin antagonism [41]. In addition, mutant ROD10 Env_Y707A_ contains a point mutation that disrupts an endocytosis motif in its cytoplasmic tail, and this is sufficient to prevent tetherin antagonism [35, 37]. We confirmed that both of these Env variants lacked the ability to counteract tetherin, since they could not stimulate the release of HIV-1 Gag-Pol virus-like particles (VLPs) from tetherin-expressing cells (Fig. 1a).

To examine whether the two mutants Envs were still capable of binding to tetherin, we created GFP-tagged versions of all three Env proteins to facilitate co-immunoprecipitation assays using anti-GFP antibodies. As expected, no tetherin was immunoprecipitated when it was co-expressed with GFP alone, or when an untagged ROD10 Env was used. However co-expression of tetherin with a GFP-tagged ROD10 Env allowed its pull-down (Fig. 1b). In contrast, the ROD14 Env mutant did not interact with tetherin. Interestingly, despite its complete lack of tetherin antagonism, the ROD10 Env_Y707A_ mutant was still able to immunoprecipitate some tetherin, and when the lower cellular levels of the ROD10 Env_Y707A_ protein were taken into consideration, it was found to be 67% (n = 3 experiments) as efficient at immunoprecipitating tetherin as the WT ROD10 Env (data not shown). These differences may be sufficient to account for its lack of anti-tetherin activity or, alternatively, this may result from some other characteristic of the Y707A mutant, such as being present in a different cellular localization than the WT Env, or because the loss of its endocytosis signal makes it unable to remove tetherin from the cell surface, as we and others have previously reported [29, 37].

### The extracellular domain of HIV-2 Env is not sufficient for anti-tetherin activity

**Fig. 2:**
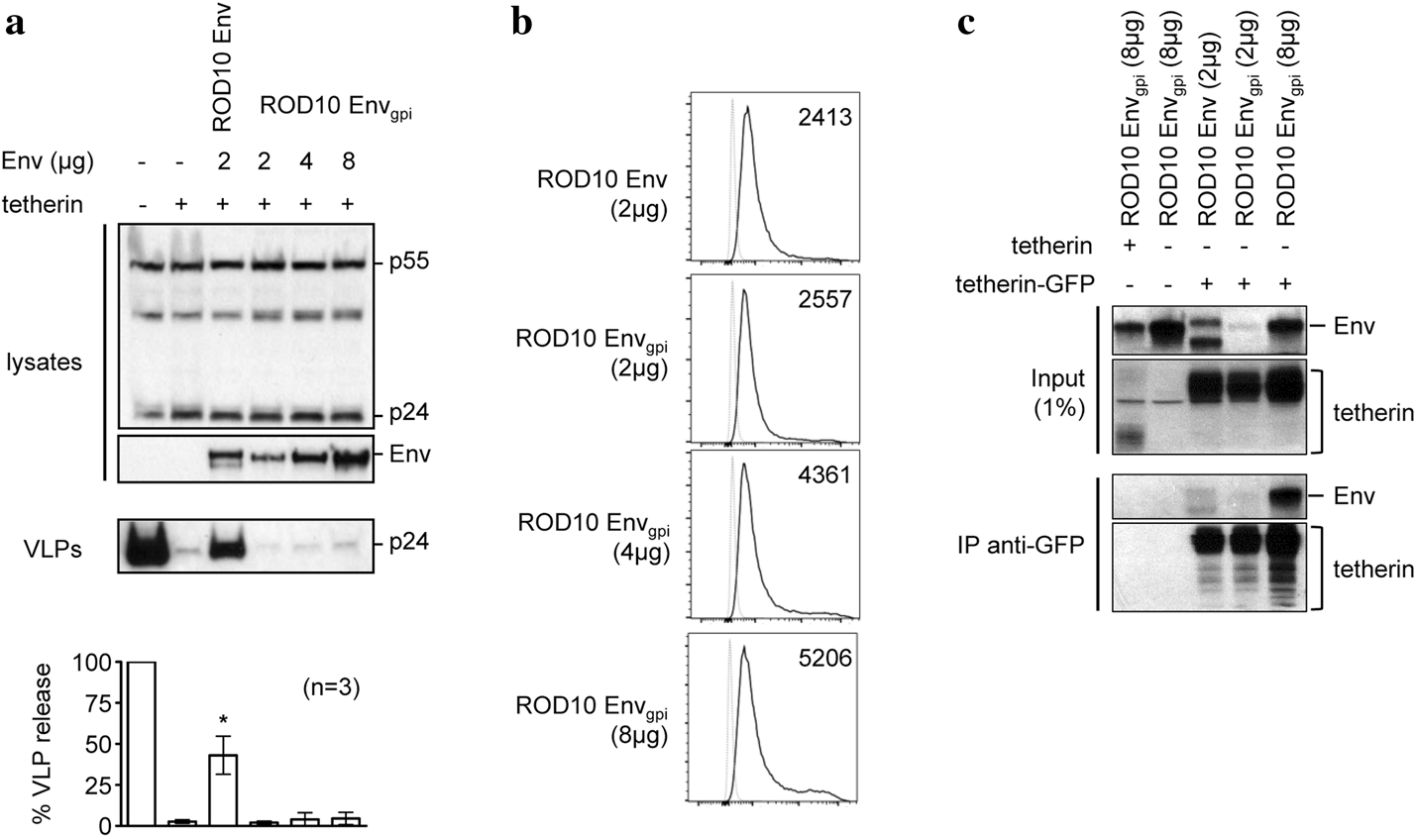
The HIV-2 Env ectodomain is not sufficient to overcome tetherin. **a** HIV-1 VLPs were produced in the presence of the WT ROD10 Env, or increasing amounts of ROD10 Env_gpi_ (2, 4, 8 μg). VLP release was analyzed by Western blotting and results normalized to the no tetherin control, for n = 3 independent experiments, *p* < 0.05 (*). **b** Cell surface expression of ROD10 Env_gpi_ was confirmed by flow cytometry. The *light grey line* represents cells stained with secondary antibody only, the *dark line* represents the indicated ROD10 Env variant. Mean fluorescence intensity (MFI) for each panel is shown. **c** 293T cells were co-transfected with tetherin or GFP-tagged tetherin, together with ROD10 Env or ROD10 Env_gpi_ at the indicated amounts. Cells were lysed 24 h later and GFP-tagged proteins were immunoprecipitated (IP) with anti-GFP MicroBeads, followed by Western blotting of the input lysates (1%) and IP products using anti-Env or anti-tetherin antibodies.

Since the interaction between tetherin and Env has been suggested to map to the extracellular domains of both proteins [35, 38], we sought to determine if the extracellular domain of the HIV-2 Env alone was sufficient for tetherin counteraction. To test this, we created a GPI anchored version of a truncated ROD10 Env protein (Env_gpi_), allowing the extracellular domain to be expressed on the cell surface in the absence of the membrane spanning domain and cytoplasmic tail. We found that expression of the ROD10 Env_gpi_ was unable to overcome tetherin restriction (Fig. 2a) despite the construct having robust cell surface expression (Fig. 2b) and being able to co-immunoprecipitate with tetherin (Fig. 2c). Together, these results indicate that while determinants sufficient to promote an Env-tetherin interaction are present in the ectodomain of Env, the membrane-spanning and/or intracellular domains of the protein are also required for functional tetherin antagonism. Such a requirement could reflect an interaction of this domain with an additional cellular partner, or the need for signals enabling co-localization of Env with tetherin at the cell surface, and/or endocytosis.

### An alanine motif in tetherin’s coiled-coil domain controls sensitivity to HIV-2 Env

**Fig. 3:**
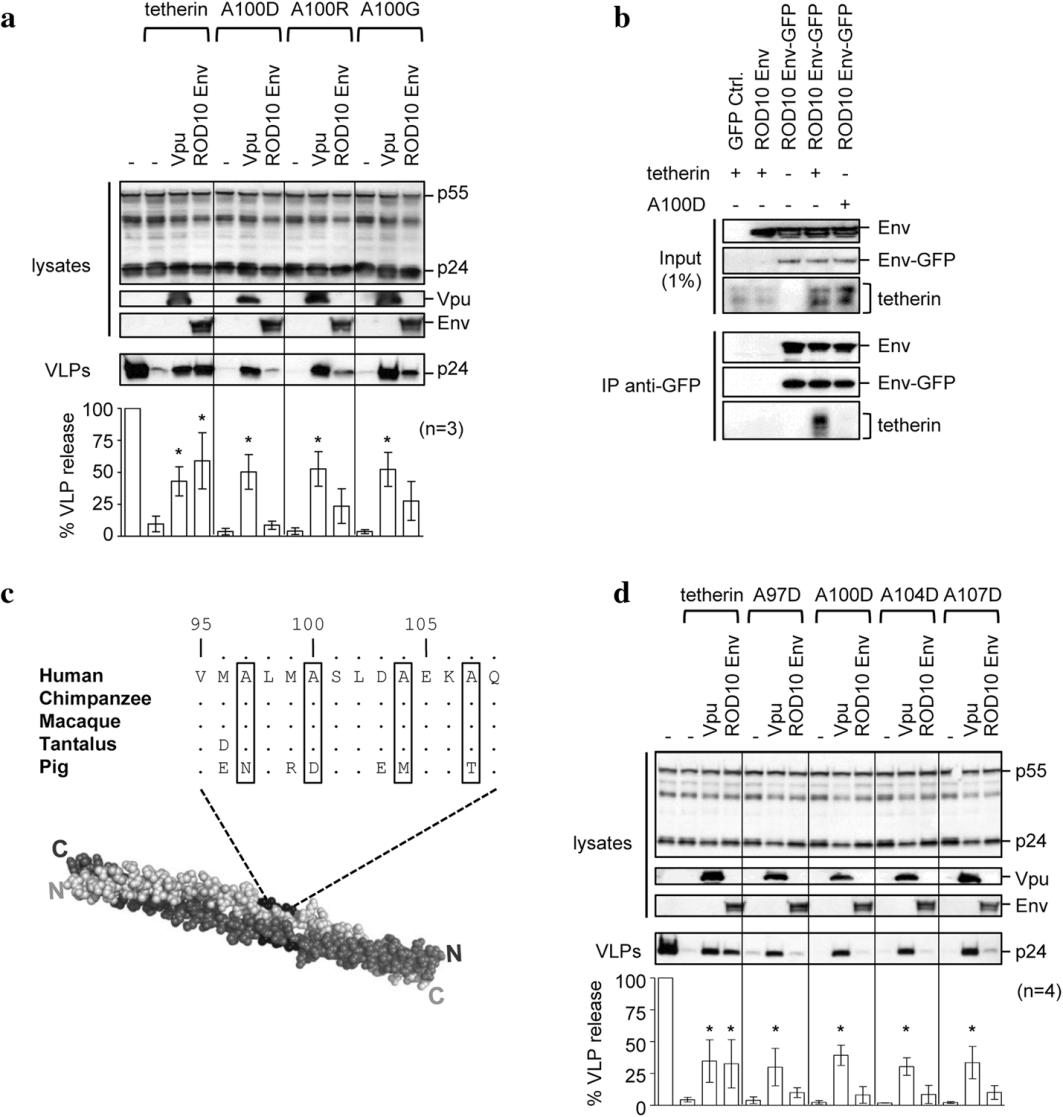
An alanine face on tetherin is required for sensitivity to HIV-2 Env. **a** HIV-1 VLPs were produced in the presence of wild-type tetherin, or mutant tetherins with single amino acid changes at position 100, together with ROD10 Env or HIV-1 Vpu. VLP release was analyzed by Western blotting and results normalized to the no tetherin control for n = 3 independent experiments, *p* < 0.05 (*). **b** The ability of ROD10 Env to bind tetherin mutant A100D was investigated by co-IP in 293T cells, using a GFP-tagged ROD10 Env. **c** The tetherin sequence from amino acids 95 through 108 is shown above the solved 3D structure of a dimer of the protein’s extracellular domain [17], created using PyMOL software (Schrödinger LLC). Four conserved alanine residues are *boxed*, and highlighted in *black* on one monomer in the 3D structure. **d** Each of the four conserved alanines were individually mutated to aspartic acid, and the ability of the resulting tetherin mutants to restrict VLP release, and be counteracted by Vpu or ROD10 Env, was assessed as previously described. Results were normalized to the no tetherin control, for n = 4 independent experiments, *p* < 0.05 (*).

We have previously shown that an alanine to aspartic acid substitution at position 100 of tetherin (A100D) makes the protein resistant to the HIV-2 Env [38] and a similarly located mutation in Tantalus monkey tetherin renders that protein resistant to SIVTan Env [42]. We next addressed whether the change to an acidic residue was important, or was it simply the loss of the specific alanine residue. Substitution of either a basic residue (arginine) or another uncharged residue (glycine) also rendered tetherin insensitive to HIV-2 Env, suggesting a specific role for the alanine residue. In contrast each tetherin mutant retained sensitivity to the HIV-1 Vpu protein (Fig. 3a).

We next asked whether the HIV-2-resistant phenotype of tetherin A100D was a result of disrupting the interaction between the two proteins. As described earlier, a GFP-tagged ROD10 Env can specifically immunoprecipitate the wild-type tetherin. However, the A100D mutant was not immunoprecipitated by the HIV-2 Env (Fig. 3b), suggesting that this substitution directly impacted the interaction between the two proteins, and accounts for the insensitivity of the mutant tetherin to HIV-2 Env.

Investigation of the sequence surrounding the A100 residue in tetherin revealed the presence of additional alanines at positions 97, 100, 104, and 107 (Fig. 3c), which are also highly conserved among primate tetherins, but absent in porcine tetherin. When mapped onto the crystal structure of a dimer of tetherin’s coiled-coil domain [17], these alanines were seen to line up on a single face, opposite to the dimerization interface, suggesting that they could be accessible to other protein partners such as HIV-2 Env. To test the hypothesis that the alanine face contributed to the interaction with HIV-2 Env, we introduced single aspartic acid substitutions at each of the four positions and tested the resulting tetherin mutants for their ability to inhibit HIV-1 VLP release, and to be counteracted by the ROD10 Env (Fig. 3d). We found that while each mutant tetherin retained the ability to restrict VLP release, and remained sensitive to Vpu, substitution of any of the alanines rendered the mutant tetherins completely resistant to ROD10 Env. These results therefore identify an alanine face on tetherin as being necessary for both the interaction with HIV-2 Env and the resulting ability of the protein to counter tetherin restriction.

### The conserved alanine motif does not render an artificial tetherin-like molecule sensitive to HIV-2 Env

Artificial tetherin (art-tetherin) contains the same structural features as native tetherin, but without the conservation of any primary sequence [20]. It is able to restrict HIV-1 release, but is resistant to both Vpu and HIV-2 Env, suggesting a sequence specific interaction between tetherin and these antagonists. However, it can be overcome by co-expression of the Ebola GP, which appears to use a different mechanism of action against tetherin [38, 43].

**Fig. 4:**
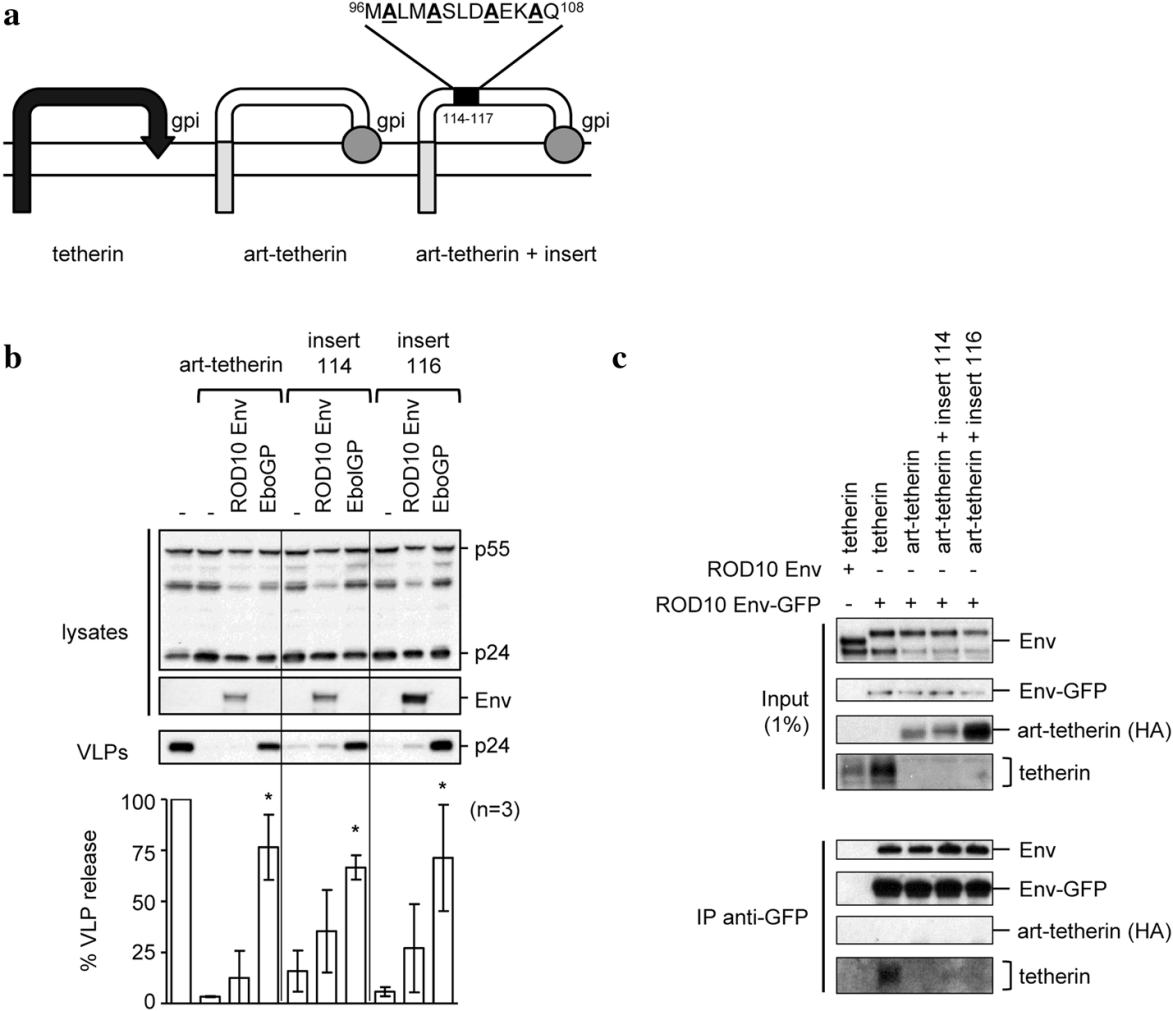
Substitution of an alanine face in art-tetherin. **a** Schematic representation of tetherin, artificial tetherin (art-tetherin), and art-tetherin derivatives with insertions of the alanine face (amino acids 95–108), at position 114–117 of art-tetherin. **b** HIV-1 VLPs were produced in 293A cells as previously described in the presence of ROD10 Env or the control Ebola GP (EboGP) expression plasmids. Additionally, art-tetherin (500 ng), insert 114, or insert 116 (1 μg each) were included and VLP release was analyzed as previously described, with results normalized to the no tetherin control, for n = 3 independent experiments, *p* < 0.05 (*). **c** 293T cells were co-transfected with the indicated artificial-tetherin variants and GFP-tagged ROD10 Env. Cells were lysed 24 h later and GFP-tagged proteins immunoprecipitated (IP) with anti-GFP MicroBeads, followed by Western blotting of the input lysates (1%) and IP products using anti-Env, anti-GFP, anti-HA (for art-tetherin) or anti-tetherin antibodies.

In order to examine whether the conserved alanine motif in the ectodomain of tetherin was sufficient to confer an interaction with HIV-2 Env, we inserted a 13 amino acid stretch of tetherin containing these alanines into the extracellular domain of art-tetherin (Fig. 4a). The sequence was inserted at four positions, each one amino acid apart, in an effort to promote exposure of the alanine face on the outside of the coiled-coil motif in at least one variant. Only two of the constructs, with inserts at positions 114 and 116, retained restrictive activity (data not shown), and these were further tested for sensitivity to both Ebola GP and ROD10 Env. As expected, both art-tetherin and the two insertional mutants remained sensitive to the Ebola GP (Fig. 4b). However, the addition of the alanine motif did not result in the acquisition of sensitivity to the HIV-2 Env, suggesting that the presence of the alanine face alone may not be sufficient to promote a functional interaction with HIV-2 Env. Furthermore, none of the art-tetherin constructs co-immunoprecipitated with ROD10 Env (Fig. 4c).

### Rates of HIV-2 replication in the presence of ROD10 and ROD14 Envs

**Fig. 5:**
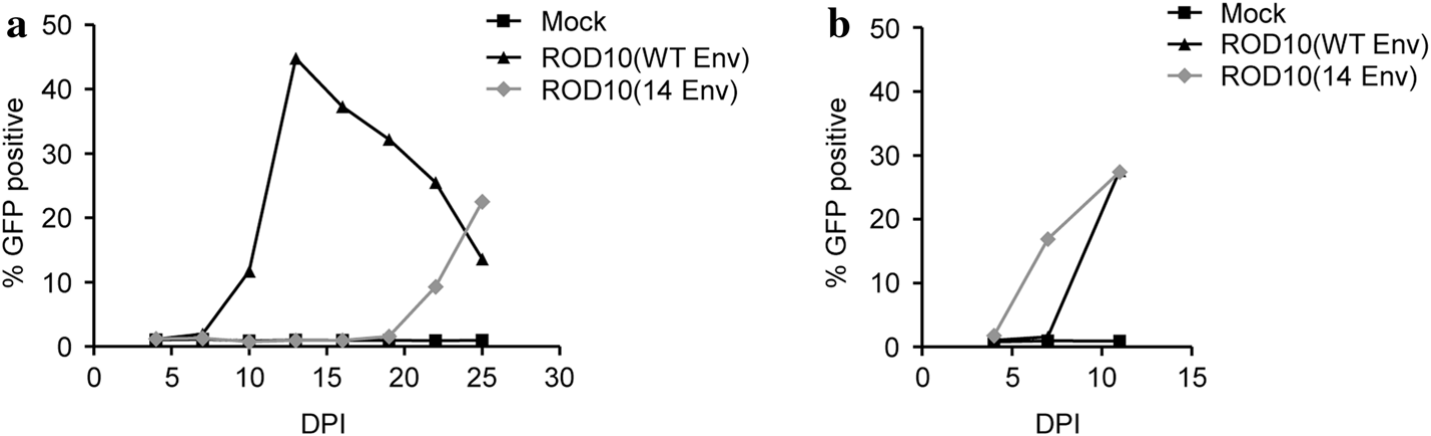
Replication of ROD10 and ROD14 Env viruses. **a** Viruses containing either the wild-type ROD10 Env [ROD10(WT Env)] or the mutant ROD14 Env protein that does not counteract tetherin [ROD10(14 Env)] were produced in 293T cells and 3 μg p27 equivalent of supernatants were used to infect 5 × 10^6^ JLTRG cells. A fraction of the cells were analyzed by flow cytometry at day 4, then every 3 days, for GFP expression. **b** Virus from day 25 in the initial infections was transferred to fresh JLTRG cells and the cells monitored by flow cytometry. Infections were stopped at day 12, when it was observed that all viruses were replicating with wild-type kinetics.

While it is well established that the presence of the HIV-2 Env can enhance virus release in one-round assays, its impact on a spreading virus replication is less well characterized, although initial studies indicated that the ROD14 virus replicated less efficiently than the ROD10 virus [40]. To investigate this further, and to ensure that any differences in viral replication were due only to defined Env mutations, we inserted the ROD14 Env mutant into the backbone of the wild-type ROD10 proviral clone to create virus ROD10(14Env). Virus stocks were created by transfecting these proviral clones into 293T cells, which do not express tetherin, allowing both wild-type and mutant viruses to be produced as virus stocks with similar titers. These stocks were then used to establish a spreading infection in JLTRG reporter cells, which express tetherin (data not shown), by assaying the cells for GFP expression as a readout of virus replication. We found that the ROD10(WT Env) virus reached a peak of infection at day 13, with about 45% of the JLTRG cells expressing GFP. In contrast, the ROD10(14 Env) mutant virus exhibited delayed replication kinetics and only began to spread through the population at day 22 (Fig. 5a).

The late rise in virus replication seen for the mutant virus suggested the possibility of the emergence of a revertant virus with increased fitness. To test this hypothesis, we took clarified supernatants from the day 25 cultures for both viruses and used them to begin new infections in fresh JLTRG cells. We observed that the passaged ROD10(14 Env) stock was now able to replicate with similar kinetics as the ROD10(WT Env) virus (Fig. 5b), suggesting that changes had occurred that increased the replicative fitness of this mutant virus, either by restoring anti-tetherin activity or through an alternate compensatory mechanism such as enhanced cell-to-cell spread.

### Revertant ROD14 Env mutants have acquired the ability to counteract tetherin

**Fig. 6:**
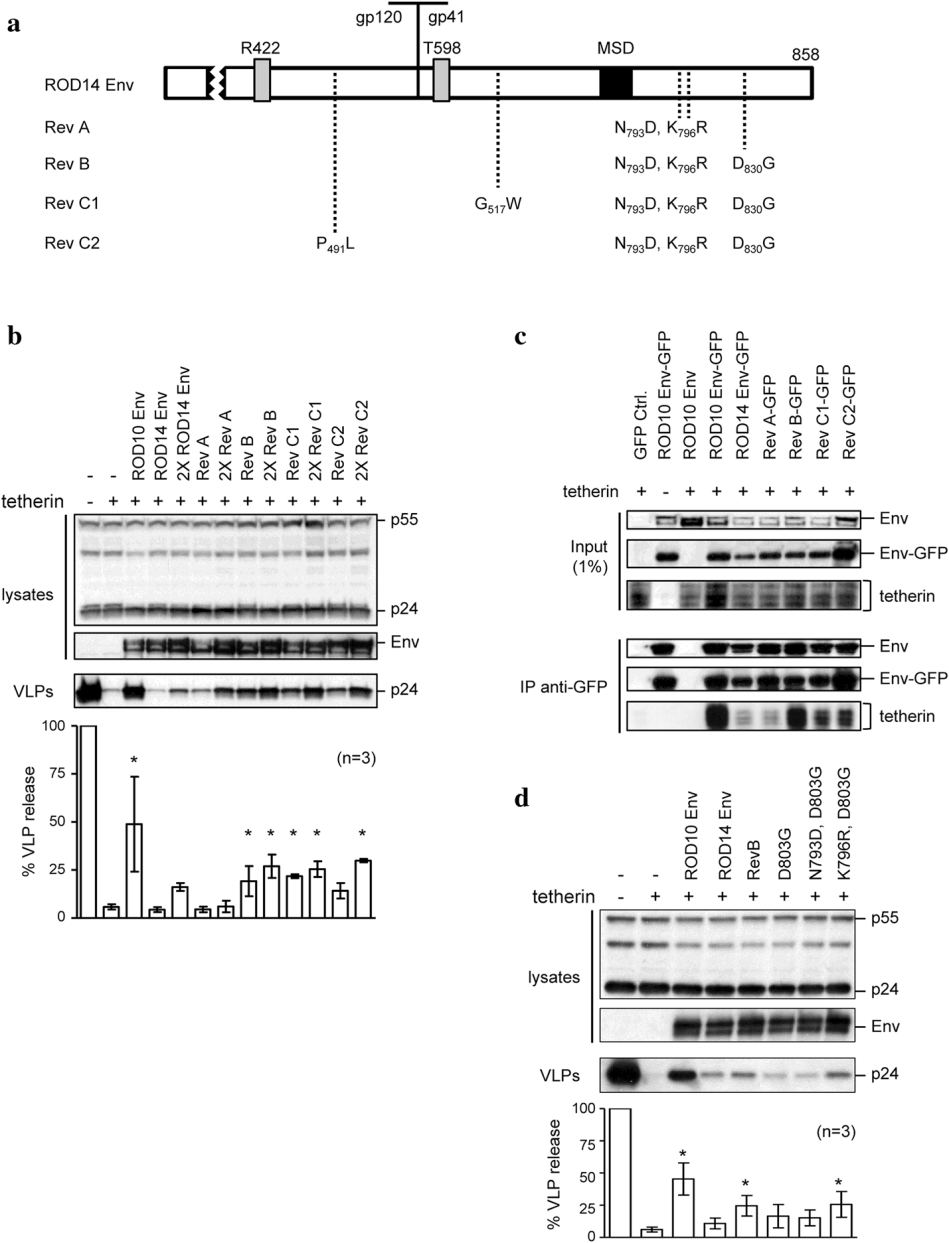
ROD14 Env reacquires anti-tetherin activity when passaged in culture. **a** Schematic of sequences in 9 independent clones isolated from ROD10(14 Env) infected JLTRG cells, with mutations shown relative to where they occur in either the gp120 or gp41 subunits. The mutations designated Rev A were found in all 9 clones, Rev B was in 7 out of 9 clones, and Rev C1 and C2 both occurred in 2 out of 9 clones each. All 9 clones retained the original ROD14 Env mutations R422 and T598, associated with loss of anti-tetherin activity. MSD is membrane-spanning domain. **b** The indicated Env clones were tested at two separate amounts, 2 and 4 μg (×2), in a VLP release assay in 293A cells in the presence of tetherin, as previously described. Results were normalized to the no tetherin control, for n = 3 independent experiments, *p* < 0.05 (*). **c** Revertant Envs were GFP-tagged and tested for their ability to co-IP with tetherin in 293T cells, as previously described. **d** Indicated Env clones, including single and double substitutions in the ROD14 Env backbone, were tested in a VLP release assay in 293A cells in the presence of tetherin. Results were normalized to the no tetherin control, for n = 3 independent experiments, *p* < 0.05 (*).

To examine the possibility that the wildtype kinetics of the passaged ROD10(14 Env) virus was due to the acquisition of an anti-tetherin activity, we isolated DNA from the second round of infections and sequenced the Env genes. For the ROD10(14 Env) stock, analysis of 9 different Env clones revealed that they all retained the signature ROD14 Env mutations previously reported to account for the protein’s loss of ability to counteract tetherin (i.e. R422 and T598). Interestingly, however, the Envs had also acquired several new mutations at other sites that differed from the parental sequence. The pattern of these mutations appeared to suggest a serial acquisition of mutations, and were designated Rev A–C (Fig. 6a).

To determine whether these mutations had produced Env proteins with anti-tetherin activity, we performed VLP release assays using expression plasmids for each of the Rev A–C Envs (Fig. 6b). We found that the first mutant in the series, Rev A, was not able to counteract tetherin. However each of the remaining proteins had acquired some activity, albeit to a lesser extent than the wild-type ROD10 Env. The ability of the mutations in Rev B to restore this activity demonstrates that mutations in the ectodomain of the ROD14 Env that prevent anti-tetherin activity can be compensated for by alterations in the cytoplasmic tail alone.

We next assessed if the anti-tetherin phenotype of this series of Envs matched their ability to interact with tetherin in a co-immunoprecipitation assay. Mirroring the virus release assay results, we found that the Rev A Env did not immunoprecipitate tetherin any more than the background levels observed with the non-functional ROD14 parent, while the Rev B, C1, and C2 Envs had all re-acquired some ability to interact with tetherin (Fig. 6c). These findings further support the idea that a direct physical interaction with tetherin is required for antagonism by HIV-2 Env and, additionally, that tetherin imposes an evolutionary pressure on HIV-2 to evolve such a tetherin counteraction strategy.

Finally, we evaluated which of the three common cytoplasmic tail mutations present in functional Rev B, C1 and C2 variants were necessary for this phenotype by evaluating single and double combinations. The lack of activity of mutant Rev A had implicated D830G as essential, but this further analysis revealed that both of the substitutions D830G and K796R were required (Fig. 6c).

### Rev B Env cytoplasmic tail changes are not sufficient to confer anti-tetherin activity

**Fig. 7:**
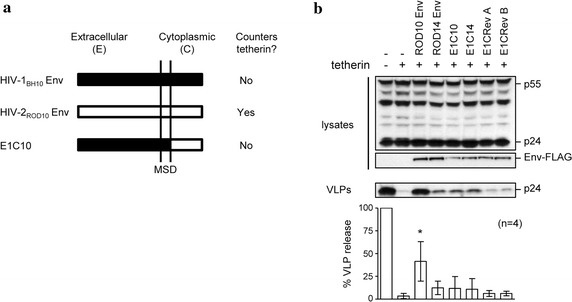
Analysis of ROD14 Env cytoplasmic tail revertants. **a** Schematic representation of the Env proteins from HIV-1 (isolate BH10), ROD10, and the chimeric protein E1C10, containing the extracellular and transmembrane domains of BH10 and the cytoplasmic domain of ROD10 Env. Similar chimeras were produced containing the BH10 extracellular domain and the cytoplasmic (C) domain of RO10, RODD14, Rev A and Rev B. MSD is membrane-spanning domain. The ability of each Env to counteract tetherin restriction is also noted [35]. **b** HIV-1 VLPs were produced in 293A cells in the presence of tetherin (100 ng), alone or together with each Env (2 μg). The percent VLP release was determined as described previously with results normalized to the no tetherin control, for n = 4 independent experiments, *p* < 0.05 (*).

We further investigated the changes that had occurred in the cytoplasmic tail of the Rev B Env by examining whether this tail alone was now capable of conferring an anti-tetherin function. Such a situation would be similar to a previous report in which a Nef-deleted SIV virus acquired mutations within the cytoplasmic tail of its Env protein, which introduced a capability to interact with, and antagonize, tetherin [44]. To examine this possibility, we created chimeric proteins containing the extracellular and transmembrane domains of HIV-1 Env, which has no anti-tetherin activity, linked to the cytoplasmic tails of either ROD10 Env (E1C10) (Fig. 7a), ROD14 Env (E1C14), or revertants A and B (E1CRev A, E1CRev B). We have previously shown that similar to the HIV-1 Env parent, the E1C10 chimera does not counteract tetherin [35]. To facilitate analysis by Western blotting, C-terminal FLAG tags were included on all proteins, including the parental ROD10 and ROD14 Envs. The resulting Env proteins were assayed for their ability to stimulate HIV-1 VLP release in the presence of tetherin (Fig. 7b). However, none of the proteins demonstrated evidence of anti-tetherin activity, indicating that the cytoplasmic tail domain of the Rev B Env was not sufficient to counteract tetherin when presented in this heterologous context.

## Discussion

The cell surface protein BST-2/tetherin exhibits several antiviral activities that derive from its ability to retain newly assembled virions at the surface [8–10]. Such tethered viruses result in a reduction in the production of cell-free virus, enhance presentation to the immune system [11–13], and lead to the induction of an antiviral state [14, 15]. To combat these activities, a range of approaches have evolved in the primate lentiviruses through adaptations in the Vpu, Nef or Env proteins of specific viruses. These antagonists act to reduce the amount of tetherin at the cell surface following intracellular sequestration [27–29, 37], displacement from the sites of viral budding [34, 45], and/or enhanced degradation [30–32]. These mechanisms appear to all involve direct or possibly indirect interactions between tetherin and the viral antagonists, since tetherin can be immunoprecipitated by each viral protein. To do this, multiple domains in tetherin are targeted, including the cytoplasmic tail by SIV Nef [26], the transmembrane region by HIV-1 Vpu [30, 46–48] and the extracellular domain by HIV-2 Env.

We confirmed a physical interaction between tetherin and HIV-2 Env by co-immunoprecipitation, and further determined that previously identified mutations in the ROD14 Env that abolished anti-tetherin activity also prevented this interaction. Furthermore, we identified an alanine face on the extracellular helical domain of tetherin as being necessary for the interaction, with substitutions at any one of four alanine residues both preventing co-immunoprecipitation and rendering human tetherin resistant to the HIV-2 Env. However inserting the alanine motif into an artificial tetherin-like molecule was not sufficient to convert the molecule to a form that could co-immunoprecipitate with the HIV-2 Env, or that was now sensitive to its antagonism, suggesting that the alanine motif is necessary, but not sufficient, for the tetherin-Env interaction.

Interestingly, an alanine face has also been implicated in the interaction between Vpu and tetherin [33, 47–49], occurring in the trans-membrane region of Vpu. Alanine faces have also been implicated in other protein–protein interactions, promoting homodimerization in other transmembrane domains [50, 51] and in receptor-agonist interactions [52, 53]. Although it was originally speculated that the alanine face in Vpu represented a direct tetherin interaction face, cysteine-scanning mutagenesis and crosslinking experiments have instead pointed to a role for the motif in maintaining the overall structure of Vpu in a form that is competent to target tetherin [34]. Therefore, while we cannot rule out that the lack of HIV-2 Env recognition of the art-tetherin molecules substituted with the alanine motif was caused by a less than optimal presentation in the context of this chimeric construct, it is also possible that the motif is not a direct interaction face, and is instead involved in maintaining the overall structure and organization of the protein’s ectodomain.

Although all of the major groups of the primate lentiviruses express anti-tetherin factors [8, 9, 21–23, 37], and their activity is easy to observe in one-round virus release assays, the importance of these activities has been less consistent in studies of virus replication. For example, studies of wild-type and Vpu-deficient HIV-1 have reported either less efficient replication for the mutant viruses [54–56], or no effect [57]. Using a similar in vitro replication assay for HIV-2 expressing the ROD10 or ROD14 Envs, we were able to observe a distinct difference in replication rates. Furthermore, the ROD14 mutant acquired wild-type replication kinetics after passage in culture, consistent with a strong selection pressure to restore, or compensate for, the loss of this activity.

Analysis of the passaged viruses revealed that they had not undergone a simple reversion back to the WT (ROD10) Env sequence. Instead, we observed both retention of the original deleterious mutations (K422R and A598T) and the acquisition of additional mutations throughout the protein. Interestingly, we found that the minimal changes required to restore anti-tetherin activity mapped to the cytoplasmic domain of the protein, specifically K796R, and D830G. Further analysis revealed that substitutions in the cytoplasmic tail also restored the ability to interact with tetherin in a co-immunoprecipitation assay, despite the presence of the ROD14 ectodomain mutations, and further suggesting that direct interactions between the two proteins is an essential part of tetherin antagonism by the HIV-2 Env.

We have previously mapped the anti-tetherin function of the HIV-2 Env to the ectodomain of the protein [35]. These findings of compensatory changes in the cytoplasmic tail suggested that either the mutations had created an additional tetherin-interacting domain in this region, or that the changes in the tail were having long-range effects on the conformation of the ectodomain and thereby restoring activity. Support for the former hypothesis comes from the characterization of Nef-deleted SIV variants that were used to infect macaques and which eventually acquired a new tetherin-binding domain in the cytoplasmic tail of Env [44]. However, chimeras created between the HIV-1 Env and these mutant cytoplasmic domains were unable to block tetherin restriction, ruling out this potential explanation. Instead, we favor a model where these mutations in the cytoplasmic tail have an ‘inside-out’ influence on the conformation of the ectodomian, and allow more permissive interactions. Long-range impacts of a cytoplasmic domain on the structure and function of a protein’s ectodomain have previously been described [58–62]. Finally, it is also possible that these cytoplasmic tail mutations could be having a more indirect effect by altering the sub-cellular localization or cell surface stability of the HIV2 Env, and thereby enhancing the activity of a less potent tetherin antagonist.

## Conclusions

BST-2/tetherin inhibits the release of budding lentiviruses. To prevent this action, HIV-2 Env sequesters tetherin in an intracellular location, in a mechanism that requires an interaction between the two proteins and which involves their ectodomains. We have mapped an alanine face in tetherin that is required, and shown that residues in both the ectodomain and cytoplasmic tail of Env can influence this interaction. HIV-2 viruses with at least one class of mutant Env protein (ROD14) reacquire this ability when passaged in culture due to second site mutations in the cytoplasmic tail, illustrating the importance of this anti-tetherin activity for viral fitness.

## Methods

### Cell lines

293T cells were obtained from the American Type Culture Collection; 293A cells were obtained from Qbiogene/MP Biomedicals (Irvine, CA, USA) and JLTRG cells [63] were obtained from the AIDS Research, Reference, and Reagent Program (ARRRP). 293A and 293T cells were maintained in Dulbecco’s modified Eagle’s medium (DMEM) (Mediatech, Herndon, VA, USA) supplemented with 10% fetal bovine serum (FBS) (Denville, Metuchen, NJ, USA) and JLTRG cells maintained in RPMI-1640 (Mediatech) supplemented with 10% FBS and 1% penicillin/streptomycin (JR Scientific, Woodland, CA, USA).

### Plasmids

Plasmid pHIV-1-pack expresses HIV-1 Gag-Pol and Rev and produces HIV-1 virus-like particles (VLPs) [35]. Plasmid pcDNA-Vphu (Vpu) encodes a human codon-optimized form of Vpu from HIV-1 isolate NL4-3 [64]. Plasmid pEboGP expresses Ebola Zaire GP-8A, the full-length form of the Ebola virus glycoprotein [38]. The HIV-2 Env expression plasmids pROD10 Env, pROD14 Env, and pROD10_Y707A_ Env have previously been described [35, 39]. C-terminal eGFP tagged versions of all Env clones were generated by 2-step PCR using plasmid pAcEGFP-N1 (Clonetech, Moutainview, CA, USA) as an eGFP template. A GPI anchored version of the extracellular domain of ROD10 Env was created by 2-step PCR to fuse residue Trp673 of Env to the GPI domain (codons 303–335) from the urokinase-type plasminogen activator receptor (uPAR) [65]. Expression plasmids for tetherin/BST-2, an eGFP-tagged tetherin/BST-2, and an artificial tetherin (art-tetherin) have been previously described [20, 29, 38]. Art-tetherin mutants containing insertions of tetherin residues 96–108 were generated by 2-step PCR. The infectious ROD10 proviral clone [36, 66] was kindly provided by Klaus Strebel (NIH). Derivatives were created containing either the ROD14 or the ROD10_Y707A_ Envs in the ROD10 backbone, using restriction sites BstAPI and BsmI at positions 8582 and 9437 in the genome. Chimeric proteins containing the transmembrane and extracellular domains of HIV-1 Env and the cytoplasmic domain of HIV-2 Env were created by 2-step PCR using the HIV-1_BH10_ proviral clone and the HIV-2_ROD10_ expression plasmids as templates, as previously described [35], and using a reverse primer that added a FLAG tag at the carboxyl terminus of the Env protein.

### Production and analysis of HIV-1 VLPs

HIV-1 VLPs were generated from 293A cells by transient transfection of pHIV-1-pack using TurboFect transfection reagent (Thermo Scientific, Glen Burnie, MD, USA), as previously described [24]. The following amounts of plasmid DNA were used per 10-cm plate of cells: 2 μg of Vpu, Ebola GP and all HIV-2 Env constructs; 100 ng of tetherin and derivatives; 500 ng of art-tetherin; 1 μg of art-tetherin mutants. Cell lysates and viral particles were collected at 24 h post-transfection and the levels of p24 proteins in both lysates and supernatants analyzed by Western blot, as previously described [29, 35, 38, 39]. The intensity of p24-reacting bands on Western blots was measured and calculated as the ratio of the signal in VLPs:lysates, normalized to the ratio for the pHIV-1-pack only control. Specific proteins were detected by Western blotting using the following antibodies: rabbit ant-HIV-1_SF2_ p24 at 1:3,000 dilution, rabbit anti-HIV-2_ST_ SU at 1:3,000 dilution, rabbit anti-tetherin at 1:10,000 dilution, and rabbit anti-HIV-1 Vpu at 1:3,000 dilution (all from ARRRP), as well as rabbit anti-GFP at 1:3,000 dilution (Abcam, Cambridge, MA, USA) and mouse anti-FLAG at 1:1,000 (Roche Applied Science, Indianapolis, IN, USA). The secondary antibodies used were HRP-conjugated goat anti-rabbit IgG at a 1:10,000 (Santa Cruz Biotechnology Inc, Santa Cruz, CA, USA) and goat anti-mouse IgG (1:10,000) (Sigma-Aldrich, St. Louis, MO, USA). Statistical analysis was performed using one-way ANOVA followed by Dunnett’s multiple comparison test from GraphPad Prism 5.0 (GraphPad Software, La Jolla, CA, USA).

### Co-immunoprecipitation

293T cells in 10-cm dishes were co-transfected by TurboFect using 200 ng of tetherin plasmid and 2 μg of the indicated eGFP-tagged HIV-2 Env plasmids or 200 ng of eGFP-tagged tetherin and either 2 μg of untagged ROD10 Env or the indicated amount of ROD10 Env_gpi_. Cell lysates were collected at 24 h post transfection and GFP pull-down assays performed using the μMACS GFP isolation kit (Miltenyi Biotech Inc., Auburn, CA, USA). Initial cell lysates (1% input) and immunoprecipitates were analyzed by Western blotting.

### Flow cytometry for Env surface expression

293A cells were in 10-cm dishes were co-transfected by Turbofect with 100 ng of an eGFP expression vector and either 2 μg of ROD10 Env or 2–8 μg amounts of ROD10 Env_gpi_. Twenty-four hours later, cells were washed 3× with PBS then blocked in 10% FBS for 30 min. Cells were then stained with HIV-2_ST_ Su antibody 1410 at a 1:300 dilution for 15 min at 4C. Cells were washed 3× with PBS then counterstained with goat anti-rabbit IgG conjugated to Alexa Fluor 647 (Invitrogen) at a 1:300 dilution for 20 min for an additional 15 min. GFP + cells (10,000 events) were analyzed on a BD FACS Canto II (BD Biosciences, San Jose, CA, USA)., and collected data was analyzed using FlowJo 6.2 software (Tree Star, Ashland, OR, USA). The mean fluorescence intensity (MFI) was determined within the software and compared to cells stained with secondary antibody alone.

### Viruses and infections

HIV-2 stocks were produced in 293T cells by transient transfection using TurboFect and 10 μg of proviral plasmids, followed by harvesting and filtration of supernatants 48 h later. Stocks were quantitated using a HIV-2 p27 ELISA kit (Zeptometrix, Buffalo, NY, USA). Infections were performed by incubating 5 × 10^6^ JLTRG cells with the equivalent of 3 μg of p27 in a total of 0.5 ml RPMI for 4 h, followed by replacement of the media with 5 ml fresh media. Every 3 days, cells were analyzed for GFP expression by flow cytometry using a FACS Canto II, with uninfected cells used to set the negative population. At each data point, 20,000 cells were collected and the data analyzed using FlowJo 6.2 software. 0.45 μm filter clarified supernatants from infected JLTRG cultures were equilibrated and added to fresh JLTRG cells for 4 h to initiate second round infections that were then tracked by flow cytometry analysis every 3 days.

Viral sequences from infected cells were obtained by isolating genomic DNA (Qiagen, Valencia, CA, USA), followed by PCR amplification using the Accuprime Taq DNA Polymerase system (Invitrogen, Carlsbad, CA, USA). The HIV-2 Env primers used were forward (GGCTTTGCACCCAACTGTTCTAAAGTAGTAGC) and reverse (CTCACTTATCGTCGTCATCCTTGTAATCCAGGAGGGCGATTTCTGCTCC), which added a FLAG tag to the cytoplasmic tail. PCR products were ligated into a TOPO-TA cloning vector (Invitrogen) and single clones selected and sequenced.
